# Study on the salivation effect of encapsulated food products containing Sichuan pepper oil

**DOI:** 10.1002/cre2.149

**Published:** 2019-01-31

**Authors:** Koufuchi Ryo, Mie Kaneko, Kei Takahashi, Hiroyuki Ono, Tomohiko Ogasawara, Michiro Abe, Ichiro Saito

**Affiliations:** ^1^ Department of Pathology Tsurumi University School of Dental Medicine Japan; ^2^ Anti‐ageing Outpatient Department Tsurumi University Dental Hospital Japan; ^3^ Sales Engineering Division Aliment Industry Co., Ltd. Japan; ^4^ Representative Director Avedes K.K. Japan

**Keywords:** *Candida* colonies, hydroxy alpha sanshool, mouth dryness, salivary secretion, Sichuan pepper, sesame oil

## Abstract

Sichuan pepper is a plant belonging to the genus *Zanthoxylum* and family rue. To evaluate whether Sichuan pepper oil boosts saliva secretion using an encapsulated food product containing the oil in subjects presenting with mouth dryness. We evaluated subjective symptoms that changed with a decrease in salivary secretion in the subjects by evaluating the number of *Candida* colonies and by conducting interviews. The study results demonstrated that salivary secretion increased by 39.4% ± 37.6% after single ingestion of the product, and an additional 8.7% ± 13.2% and 6.3% ± 31.2% following continuous ingestion over 2 and 4 weeks, respectively. These findings suggested that the product rapidly promotes and maintains salivation. Regarding the proliferation of *Candida* colonies in subjects with mouth dryness, a negative correlation was observed between *Candida* colony number and salivary secretion quantity. Additionally, interviews revealed that subjective symptoms, such as mouth dryness, discomfort and pain in the mouth, difficulty swallowing the saliva, and feeling of stickiness in the mouth, improved shortly after single ingestion of the product, and mouth dryness was reduced by continuous consumption of the product. These findings indicated that the product studied promotes rapid salivary secretion, is effective in reducing the number of oral *Candida* colonies, and improves subjective symptoms such as mouth dryness.

## 1 INTRODUCTION

Mouth dryness is characterized by abnormal dryness in the mouth and throat due to reduced salivary secretion and changed saliva quality. It is typically accompanied by oral pain, discomfort, dysgeusia, and halitosis. It significantly decreases the quality of life (QOL) by making it difficult to ingest food and to speak, in addition to causing worsening infections that increase the risk of dental caries and periodontal disease. Furthermore, mouth dryness also results in systemic diseases, such as aspiration pneumonitis (Chambers, Garden, Kies, & Martin, 2004). The incidence of mouth dryness symptoms is particularly high in the elderly (Higuchi et al., 2009). Studies in Europe and the United States revealed that approximately 29% of the elderly people have subjective symptoms of discomfort associated with mouth dryness (Sreebny & Valdini, 1988), and that approximately 16% and 25% of elderly men and women, respectively, present with mouth dryness (Osterberg, Landahl, & Hedegård, 1984). Although the precise cause of mouth dryness remains unclear, the overall consensus is that age, sex, diseases, and adverse drug reactions are all contributory factors (Ben‐Aryeh, Miron, Berdicevsky, Szargel, & Gutman, 1985; Gutman & Ben‐Aryeh, 1974; Nagler & Hershkovich, 2005; Navazesh, Mulligan, Kipnis, Denny, & Denny, 1992; Osterberg et al., 1984; Ship, Pillemer, & Baum, 2002). The effects of age and sex are particularly significant; mouth dryness increases with age, and the incidence tends to be higher in women in their 50s who are nearing menopause (Flink, 2007; Figure S1).

Approximately 8 million patients in Japan, or 25% of the total population, are affected by mouth dryness (Guggenheimer & Moore, 2003). Consequently, there has been a growing demand for an effective remedy for mouth dryness symptoms. Previous studies have evaluated food materials free of adverse reactions that can improve mouth dryness, such as astaxanthin, soy isoflavone, catechin, and coenzyme Q10 (Ryo et al., 2011; Ryo et al., 2014; Yamada et al., 2010; Yamamoto et al., 2004). Here, we focused on the effects of Sichuan pepper (*Zanthoxylum bungeanum*).

Sichuan pepper is a plant belonging to the genus *Zanthoxylum* and family rue. Its common Chinese name is “huajiao” and is cultivated mainly in the Hebei and Shanxi provinces of China, as well as in Southeast Asian countries. The fruit and pericarp of the Sichuan pepper have a characteristic pungent flavor called “maa (麻)” and are used as spices in various Chinese dishes (Zhu et al., 2011). In China, Sichuan pepper has been traditionally used as an herbal medicine due to the belief that it promotes stomach health, reduces inflammation, relieves pain from dental caries, and relieves abdominal pain from ascariasis (Gong et al., 2009). We found that Sichuan pepper not only has a good taste but also has an adequate pungent flavor that can promote salivation.

Therefore, we conducted a detailed examination of this plant. The results showed that the oil‐soluble fraction extracted from Sichuan pepper (Sichuan pepper oil) has a very strong salivation effect. Therefore, we prepared an encapsulated food product containing Sichuan pepper oil (Aliment Industry Co., Ltd., Yamanashi, Japan) for individuals with mouth dryness symptoms. For examining the usefulness of the product, we evaluated subjective symptoms based on the level of saliva secretion, number of oral *Candida* colonies, and responses in interviews.

### 1.1 Subjects and methods

#### 1.1.1 Subjects

This study was implemented after obtaining approval of the ethical review committee of Tsurumi University School of Dental Medicine (date: 27 May 2014) and complied with the ethical standards of the Declaration of Helsinki. The physician explained the study purpose and method and obtained written consents from the subjects. The subjects were male and female nonsmokers (age 20–80 years when obtaining written consent) who presented with subjective symptoms of mouth dryness to the Dry Mouth Outpatient Department at Tsurumi University Dental Hospital. Applicants who met any of the following criteria were excluded (any internal medicines that had been used to date were continued without changing the dosage): (1) individuals with medical history of salivary gland disease within 6 months before the start of the study or who are currently under treatment; (2) those currently on medications or have consumed them within 1 month of starting the study, which could affect salivary secretion; (3) those who are currently participating in other studies or have participated within the past 3 months (those who participated in studies concerning long‐term carry‐over effects and safety were limited to within 6 months); and (4) those judged to be inappropriate for participation due to other reasons by the physician.

This study is registered with the University Hospital Medical Information Network Clinical Trials Registry (UMIN‐CTR; Registration number: UMIN000027493).

#### 1.1.2 Product

The product was a seamless capsule (7 mm diameter, 155 mg weight; Aliment Industry Co., Ltd.) containing Sichuan pepper oil, white sesame oil, menthol, and Sicilian lemon oil. The subjects ingested three capsules before every meal for 4 weeks.

#### 1.1.3 Study method

Evaluations were performed by measuring the salivary secretion using the Saxon test (g/2 min) and counting the number of *Candida* colonies on the dorsum of the tongue. Subjective symptoms were evaluated by administering questionnaires and conducting interviews.

##### Saxon test (stimulated salivary secretion)

A sterile gauze pad (Tamagawa‐Eizai Co., Ltd.) with dry weight was placed in the mouth of each subject who was then asked to chew at a rate of 1 bite for 2 min. The weight of the wet saliva‐absorbing gauze pad was then measured using an electric balance to calculate the stimulated salivary secretion. Measurement time points were before ingestion, after single ingestion, and after 2 and 4 weeks of continuous ingestion.

##### Oral *Candida* colony test

Samples were collected by swabbing the dorsum of the tongue 10 times with a cotton bud, smearing the sample on CHROMagar medium, incubating at 37°C for 48 h and then counting the number of *Candida* colonies. Measurement time points were before ingestion, after single ingestion, and after 2 and 4 weeks of continuous ingestion.

##### Evaluation of subjective symptoms by questionnaires and interviews

A questionnaire entitled “Interview Sheet on Oral Condition” (Table 1), consisting of 30 questions regarding mental, physical, and oral conditions, was administered to each subject. The investigator interviewed each subject regarding subjective symptoms and then evaluated the severity of the symptoms on a five‐grade scale before ingestion, after single ingestion, and after 2 and 4 weeks of continuous ingestion. On the basis of the answers to the first nine questions directly related to mouth dryness symptoms, the product was expected to act rapidly on symptoms. Therefore, we evaluated the severity of oral symptoms after single ingestion. In addition, a free‐answer section was provided in the questionnaire.

**Table 1:** “Interview Sheet on Oral Condition” questionnaire

Interview: before ingestion, after single ingestion, and after 2 and 4 weeks of continuous ingestion	
1	Drying of the mouth
2	Pain and discomfort in the mouth
3	Feeling of insufficient salivary secretion
4	Difficulty swallowing saliva
5	Feeling of stickiness in the mouth
6	Saliva without appear sustainably
7	Feeling of stimulation in the mouth
8	Pain in the tongue
9	Difficulty conversing
Interview: before ingestion and after 2 and 4 weeks of continuous ingestion	
10	Food without being eaten
11	Difficult to chew any food
12	Difficult to swallow any food
13	Difficulty in eating
14	Cannot get along with a person because of constantly being anxious about the mouth
15	Worrying about something wrong with the mouth and teeth
16	Worrying about eyes to the mouth
17	Worry about the mouth and not relaxed when eating in public
18	Have sensitive teeth and mouth
19	Worrying about water at the time of a meal
20	Strange taste
21	Breathing with mouth
22	Worrying about halitosis
23	Food stuck between the teeth
24	Shaking of teeth
25	Swollen gums
26	Rough lips
27	Worrying about inferiority of the corners of the mouth
28	Worrying about wrinkles of the whole face
29	Worrying about wrinkles around the mouth

*Note*. The severity of subjective symptoms of each subject was evaluated by the following five‐grade scale by the investigator via an interview. Five‐grade scale: 0: remarkable effective; 1: effective; 2: slightly effective; 3: neutral; 4: worse.

### 1.2 Statistical analysis

Figure data are shown as the mean ± standard error. The Student's *t* test was used for all tests. Significance level was *P* < 0.05.

## 2 RESULTS

### 2.1 Subjects

A total of 10 male and female subjects who were judged to be competent by the study physician were reviewed. However, one male subject was excluded from this study because he was 85 years old. Thus, a total of nine female subjects (mean age, 68.3 ± 9.5 years) were enrolled in this study.

### 2.2 Salivary secretion

**Figure 1:**
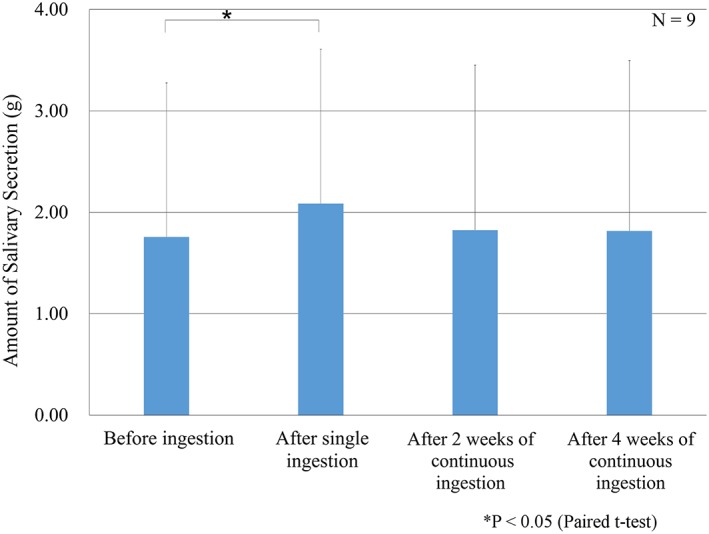
Changes in the amount of salivary secretion (gram). Salivary secretion quantity increased significantly after a single ingestion of the product *p* < 0.05 and it continued to increase after continuous ingestion for 2 and 4 weeks compared with that before ingestion. Each saliva secretion quantity was measured 3 min after 3 grains/time ingestion of the product

As shown in Figure 1 based on the amount measured before ingestion, saliva secretion showed an increasing trend (39.4% ± 37.6%, 8.7% ± 13.2%, and 6.3% ± 31.2% after single ingestion and 2 and 4 weeks of consecutive ingestion, respectively). In particular, the amount of saliva secretion after single ingestion was significantly increased (*P* < 0.05).

### 2.3 Number of *Candida* colonies

**Figure 2:**
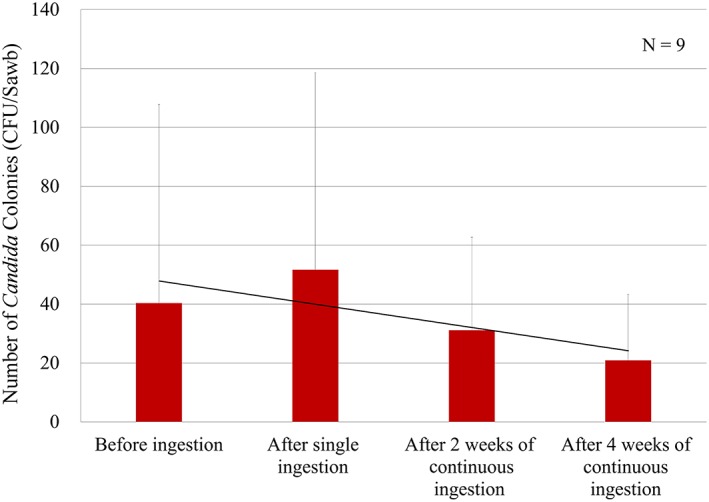
Changes in the number of Candida colonies (cfu/swab). The number of oral Candida colonies decreased continuously after ingesting the product for 4 weeks

As shown in Figure 2, there was a transient increase in the number of oral *Candida* colonies after single ingestion, but this number markedly decreased following continuous ingestion for 2 and 4 weeks. There was a negative correlation between the number of *Candida* colonies and salivary secretion, but this association was not statistically significant.

### 2.4 Subjective symptoms by questionnaires and interviews

**Figure 3:**
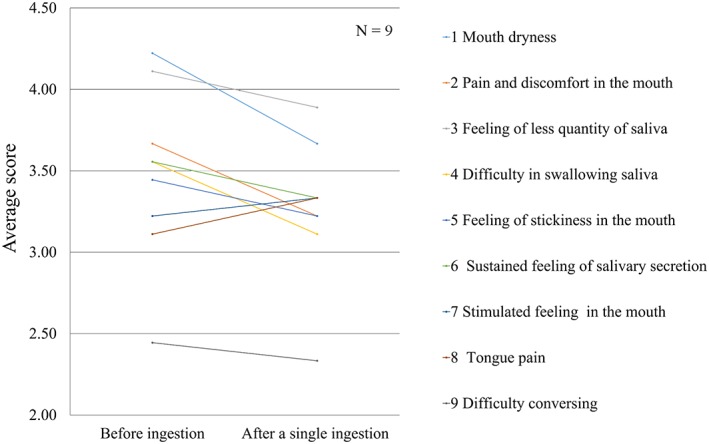
Changes in average scores for subjective symptoms after a single ingestion. Subjective symptoms such as mouth dryness, pain and discomfort in the mouth, difficulty in swallowing saliva, feeling of something sticky in the mouth, sustained feeling of salivary secretion, and difficulty in conversing improved immediately after a single ingestion

**Figure 4:**
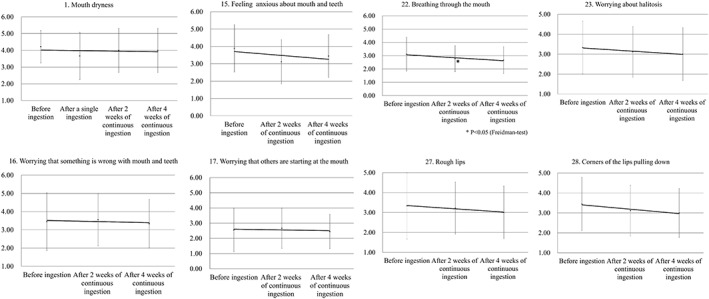
Changes in the average scores for subjective symptoms after continuous ingestion. The conditions such as mouth dryness, feeling anxious about mouth and teeth, worrying about something wrong being with the mouth and teeth, worry about eyes to the mouth, breathing through the mouth, worrying about halitosis, lips getting rough, and pulling down of the corners of the lips improved after continuous ingestion for 2 and 4 weeks

Evaluation of subjective symptoms indicated that the treatment was effective in four subjects, slightly effective in four, and neutral in one. Therefore, the combined effectiveness rate for the treatment being effective and slightly effective was 88.8%. When evaluating subjective symptoms after single ingestion, an improvement was observed in seven of the nine conditions (77.8%), including mouth dryness, pain and discomfort in the mouth, difficulty in swallowing saliva, feeling of stickiness in the mouth, sustained feeling of salivary secretion, and difficulty in conversing (Figure 3). After continuous ingestion for 2 and 4 weeks, an improvement was observed for the conditions of feeling anxious about the mouth and teeth, breathing through the mouth, halitosis, rough lips, and drooping mouth corners (Figure 4). In addition, there was relief from symptoms of mouth dryness, such as tongue pain, thirst, and feeling of salivary secretion.

### 2.5 Overall rating (utility evaluation)

Regarding the overall use of the product, we conducted an interview to comprehensively evaluate the changes in salivary secretion, number of *Candida* colonies, rating of subjective symptoms by questionnaire and presence/absence of adverse events (subjective/objective symptoms). The results demonstrated that the product was very useful in none of the subjects, useful in four, slightly useful in three, neutral in two, and not useful in none. Thus, the overall rate for the usefulness of the product was 44.4% and that for the product being somewhat useful or more was 77.7%. The product was assessed as being neutral by two subjects, one of whom demonstrated improvements, such as decreased number of *Candida* colonies and reduced difficulty in eating food. However, no improvement was observed in the symptom of angular stomatitis. In the other subject, salivary secretion increased, but there were no changes in the number of *Candida* colonies or subjective symptoms.

### 2.6 Adverse events

No adverse events were associated with the ingestion of the product in any subject.

## 3 DISCUSSION

This study focused on the salivation effects of Sichuan pepper to improve subjective symptoms of mouth dryness. Sichuan pepper contains sanshool derivatives, such as hydroxy‐α‐sanshool, which causes strong tingling sensation and numbness and pungent components, such as sansho amide. The product was prepared by diluting a stock solution of Sichuan pepper oil, which was cold‐pressed and extracted from a mixture containing dried pericarp of Sichuan pepper. Sesame oil was used as a diluent to adjust the concentration and reduce the degree of stimulation caused by the pungent components of the plant. We also used a unique masking technique of adding Sicilian lemon oil as a flavoring ingredient to make the product more palatable, and encapsulated the mixture in a seamless capsule.

We asked the subjects with mouth dryness symptoms to consume the product for 4 weeks to examine its effects on salivation. The treatment of mouth dryness conventionally includes symptomatic treatment using artificial saliva, moisturizing gel to protect the dried oral mucosa, and pharmacotherapy using a salivary secretion medicine or myofunctional therapy. In particular, for patients with Sjögren syndrome, presenting with decreased salivary secretion due to the impairment of the salivary gland, a salivary secretion medicine may be prescribed as part of the pharmacotherapy to promote salivary secretion by stimulating the parasympathetic nervous system via muscarinic receptors in the salivary glands. However, because some patients experience adverse reactions to salivary secretion medicines, new products that can promote salivary secretion are warranted (Table S1).

**Figure 5:**
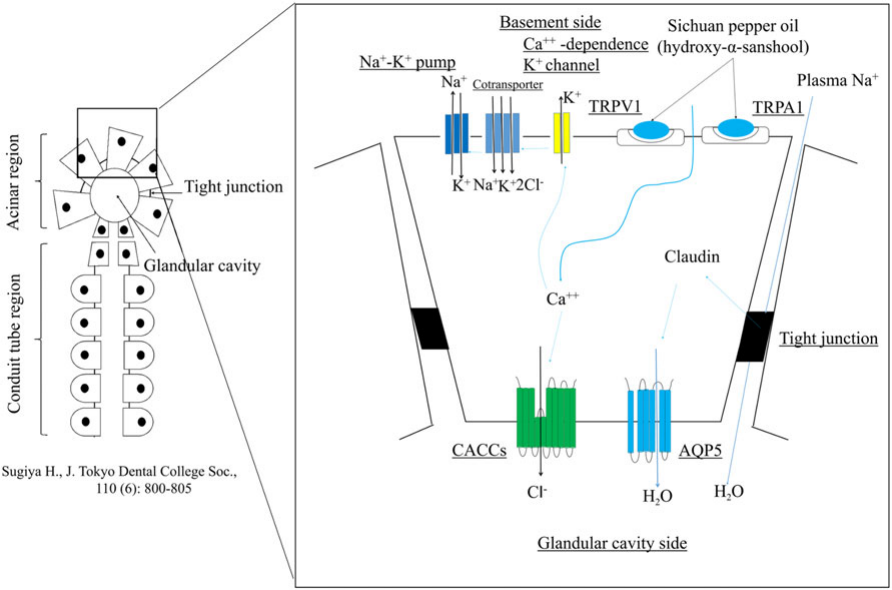
Salivary gland composition and mechanism of water secretion. Sichuan pepper oil increases intracellular Ca^++^ concentration by transporting extracellular Ca^++^ into cells via TRPV1 and TRPA1. Then, the Ca^++^‐dependent K^+^ channel opens to transport intracellular K^+^ out of cells, and conjugated Na^+^/K^+^/2Cl^−^ cotransporter is activated to transport K^+^ back into cells, as Na^+^ and Cl^−^ flow into cells simultaneously. The increased intracellular Ca^++^ also opens the Ca^++^‐dependent Cl^−^ channelto bring intracellular Cl^−^ out of cells into the glandular cavity and plasma Na^+^ is attracted toward the glandular cavity. Thus, osmotic pressure increases in the glandular cavity; hence, water from the cells and from the plasma is secreted in the vessels. Claudin that increases as TRPV1 is activated acts on the aquaporin in the water channel to secrete water into the glandular cavity

Saliva is produced by acinar cells, and some of its components are resorbed into the blood while it passes through the vessels. Blood components flow directly into the vessels, and saliva is finally secreted into the mouth (Figure 5; Sugiya, 2010). Our study focused on the salivation effects of Sichuan pepper and showed that the Sichuan pepper‐containing product promoted salivary secretion. On the basis of the results of previous studies, we hypothesized that a strong association exists between the hydroxy‐α‐sanshool contained in the pericarp of Sichuan pepper, which is an agonist of pain‐integrating cation channels (TRPV1 and TRPA1) that promote high Ca permeability in the salivary gland, and increased salivary secretion (Bader, Stark, Dawid, Lösch, & Hofmann, 2014; Tominaga, 2011). As shown in Figure 5, Sichuan pepper oil (hydroxy‐α‐sanshool) increases intracellular Ca^++^ concentration by transporting extracellular Ca^++^ into cells via TRPV1 and TRPA1. Then, Ca^++^‐dependent K^+^ channels in the basal‐lateral membrane export intracellular K^+^ out of the cells, and the conjugated Na^+^/K^+^/2Cl^−^ cotransporter is activated to transport K^+^ back into the cells, while Na^+^ and Cl^−^ flow into the cells at the same time. Na^+^ is transported out of the cells by Na^+^–K^+^ pumps on the basal‐lateral membrane. Increased intracellular Ca^++^ concentrations cause opening of Ca^++^‐dependent Cl^−^ channels in the membrane of the glandular cavity, which export intracellular Cl^−^ out of the cells and into the glandular cavity. It has been suggested that Cl^−^ is exported to create an electrically negative condition because plasma Na^+^ is attracted towards the glandular cavity via the paracellular transport pathway. This increases the osmotic pressure in the glandular cavity, and intracellular water and water from the plasma are secreted into the vessels via the paracellular transport pathway (Melvin, Yule, Shuttleworth, & Begenisich, 2005; Sugiya, 2011). It has also been reported that water secretion requires claudin, a tight‐junction protein, and it increases with TRPV1 activation (Bautista et al., 2008; Shin, Kim, & Park, 2016). Claudin acts on aquaporin to secrete water into the glandular cavity via the transcellular transport system (Michikawa & Fujita‐Yoshigaki, 2008; Shin et al., 2016).

The functional ingredient contained in the product was hydroxy‐α‐sanshool(Bautista et al., 2008). It is known that ingesting Sichuan pepper causes a tingling sensation in the tongue because hydroxy‐α‐sanshool induces Ca^++^ inflow into the cells via the pain‐integrating cation channels TRPV1 and TRPA1, which generate action potentials (Bader et al., 2014; Koo et al., 2007). Additionally, it has been suggested that this tingling sensation is induced as nerve cells are activated due to the blocking of intracellular pH‐ and anesthetic‐sensitive two‐pore K^+^ channels (Bautista et al., 2008). Our product was also characterized by its excellent digestibility, which was achieved by maintaining the function of hydroxy‐α‐sanshool while reducing the tingling sensation caused by it using a unique masking technique. Virtually, no symptoms of irritation were reported in this study; hence, we concluded that the product could be safely ingested. Furthermore, the product had a salivation‐promoting effect and improved subjective symptoms, such as mouth dryness, feelings of stickiness in the mouth, and discomfort caused by decreased salivary secretion. Bader et al. (Tominaga, 2011) reported that the tingling sensation caused by hydroxy‐α‐sanshool is associated with salivary activity when examining salivary secretion in healthy volunteers (*n* = 8). Their results demonstrated that compared with the control (water), saliva secretion increased by 75% with hydroxy‐α‐sanshool, 25% with hydroxy‐β‐sanshool, and 44% with a mixture of both the compounds (9.1% hydroxy‐α‐sanshool + 2.5% hydroxy‐β‐sanshool). On the basis of these findings, the study suggested that a chemical structure with at least one *cis*‐configured double bond, similar to that found in hydroxy‐α‐sanshool, is required to induce a strong salivation effect and that salivary secretion is decreased by a chemical structure with an all‐*trans*‐configured double bond, similar to that found in hydroxy‐β‐sanshool (Figure S2; Bader et al., 2014).

Saliva is secreted through the gustatory salivary reflex circuit when the TRPV1 receptor in gustatory cells is activated by a soluble stimulant or gustatory sensation. The basic reflex circuit significantly activates communication between the nucleus of the solitary tract and salivatory nucleus in the brainstem region (Matsuo, 2006). The periodontal membrane and oral mucosa are stimulated by chewing food to promote salivary secretion by activating the salivatory nucleus in the brainstem region or gustatory center, as well as the sympathetic nerves in the salivary gland (Matsuo, 2006). The product examined in this study contained Sichuan pepper oil, along with menthol and flavoring ingredients, such as Sicilian lemon oil, to modify the taste for better digestibility. Tateyashiki et al. (Tateyashiki, Imaizumi, & Mori, 1992) reported that salivary secretion is increased by smelling lemon peel, suggesting that the increase in salivary secretion in this study was induced by the combined action of the ingredients (hydroxy‐α‐sanshool, limonene, and Sicilian lemon oil) during chewing. Additionally, sesame oil in the product formed an anatomical barrier on the epithelium of the oral mucosa, which helped to prevent oral dryness and adhesion/invasion of *Candida* (Table S1, Figure S3).

In our study, salivary secretion was promptly induced after single ingestion of the product. The amount of secreted saliva was significantly increased, and the saliva tended to be secreted in a continuous manner as the product was subsequently ingested for 4 weeks. Although no statistically significant correlation was found between the number of *Candida* colonies and salivary secretion, changes in the number of *Candida* colonies demonstrated a negative correlation with salivary secretion. Mori et al. (Mori, 2012) reported that decreased saliva flow causes symptoms associated with oral dryness, such as atrophy of the oral mucosa, dental caries, dysphagia, and fungal infection. However, salivary secretion can be increased by improving the oral environment, and as a consequence, the number of *Candida* colonies can be reduced and symptoms of oral dryness can be improved (Mori, 2012). We demonstrated that subjective symptoms, such as oral dryness, discomfort and pain in the mouth, difficulty in swallowing saliva, and feeling of stickiness in the mouth, quickly improved after single ingestion of the product. Although no statistically significant correlation was found between salivary secretion and symptom relief, from a clinical viewpoint, the significant increase in salivary secretion relieved the symptoms associated with oral dryness. Furthermore, a feeling of salivary secretion was maintained; hence, continuous ingestion of the product improved the symptoms associated with oral dryness. No adverse events were attributable to the product, thereby confirming its safety.

Our findings suggested that oral dryness can be relieved by continuous ingestion of the product before meals, thereby improving QOL.

## 4 CONCLUSION

Our study demonstrated the salivation effect of an encapsulated food product containing Sichuan pepper oil in subjects presenting with subjective symptoms of mouth dryness and reduced the number of oral *Candida* colonies.

## CONFLICT OF INTEREST

This study did not receive any specific funding. The authors have stated explicitly that there are no conflicts of interest related to this article.

## Supporting information

Table S1. Composition of the encapsulated food product containing sichuan pepper oil

Figure S1. Causes of mouth dryness. Mouth dryness is caused by various factors such as physiological, underlying disorders, and lifestyle habits.

Figure S2. The effect of hydroxy‐α‐sanshool on salivary secretion. The hydroxy‐α‐sanshool with at least one cis‐configured double bond induces a stronger salivation effect than the hydroxy‐β‐sanshool with all‐trans‐configured double bond (8 healthy volunteers).

Figure S3. Improvement in the dryness of the oral cavity (inference). When chewing the product, ingredients present in the product such as hydroxy‐α‐sanshool, limonene, and Sicilian lemon oil act comprehensively to promote salivary secretion. Chewing also leads to salivary secretion by stimulating the salivary glands. The sesame oil forms an anatomical barrier on the epithelium of the oral mucosa to prevent oral dryness and adhesion/invasion of Candida. Thus, the subjective symptoms, such as oral dryness, sticky sensation, and discomfort/pain in the mouth after ingestion are ameliorated quickly by the product.
