# Supplementary material for: Study on the salivation effect of encapsulated food products containing Sichuan pepper oil
Source: Clin Exp Dent Res. 2019 Jan 31;5(1):7–13. doi: 10.1002/cre2.149 (PMC6392883; doi:10.1002/cre2.149)
Supplement: Supplementary file 1 — Table S1. Composition of the encapsulated food product containing sichuan pepper oil [file CRE2-5-7-s001.doc]

Supplementary Table S1. Composition of the encapsulated food product containing sichuan pepper oil

| Function | Salivation secretion | | Oral mucosa protection, oral moisturizing , oral dryness  prevention | |
| --- | --- | --- | --- | --- |
| Category | Drug | The product  (Sichuan pepper oil) | Drug | The product  (Sichuan pepper oil) |
| Mode of action | Stimulating parasympathetic nervous system via muscarinic M3 receptor | Stimulating parasympathetic nervous system via nociceptor receptor | Preventing dryness of oral mucosa | Forming an anatomical barrier on the epithelium of the oral mucosa |
| Active ingredient | Pilocarpine, Cevimeline | Hydroxy-α-sanshool | Sodium chloride  Potassium chloride  Calcium chloride hydrate  Magnesium chloride  Dipotassium hydrogen  monophosphate | Sesame oil |
| Formulation | Tablet, Capsule | Soft capsule | Spraying aerosol agent | Soft capsule |
| Usage/dosage | Oral administrating 3 times a day daily after each meal | Chewing and ingesting approximately 2-3 grains/time, when worrying about mouth dryness | Spraying 4-5 times a day in the mouth for 1-2 seconds at a time. Adjust according to the symptoms as appropriate | Chewing and ingesting approximately 2-3 grains/time, when worrying about mouth dryness |
| Target | ●Improvement of xerostomia associated with radiotherapy of the head and neck  ●Improvement of xerostomia in patients with Sjogren's syndrome | Ameliorating oral dryness, a feeling of stickiness and discomfort/pain in the mouth | ●Xerostomia due to Sjogren's syndrome  ●Xerostomia on salivary gland injury due to irradiation of the head and neck | Oral mucosa barrier creation and moisturisation |
