# Supplementary figures and images for: Study on the salivation effect of encapsulated food products containing Sichuan pepper oil
Source: Clin Exp Dent Res. 2019 Jan 31;5(1):7–13. doi: 10.1002/cre2.149 (PMC6392883; doi:10.1002/cre2.149)

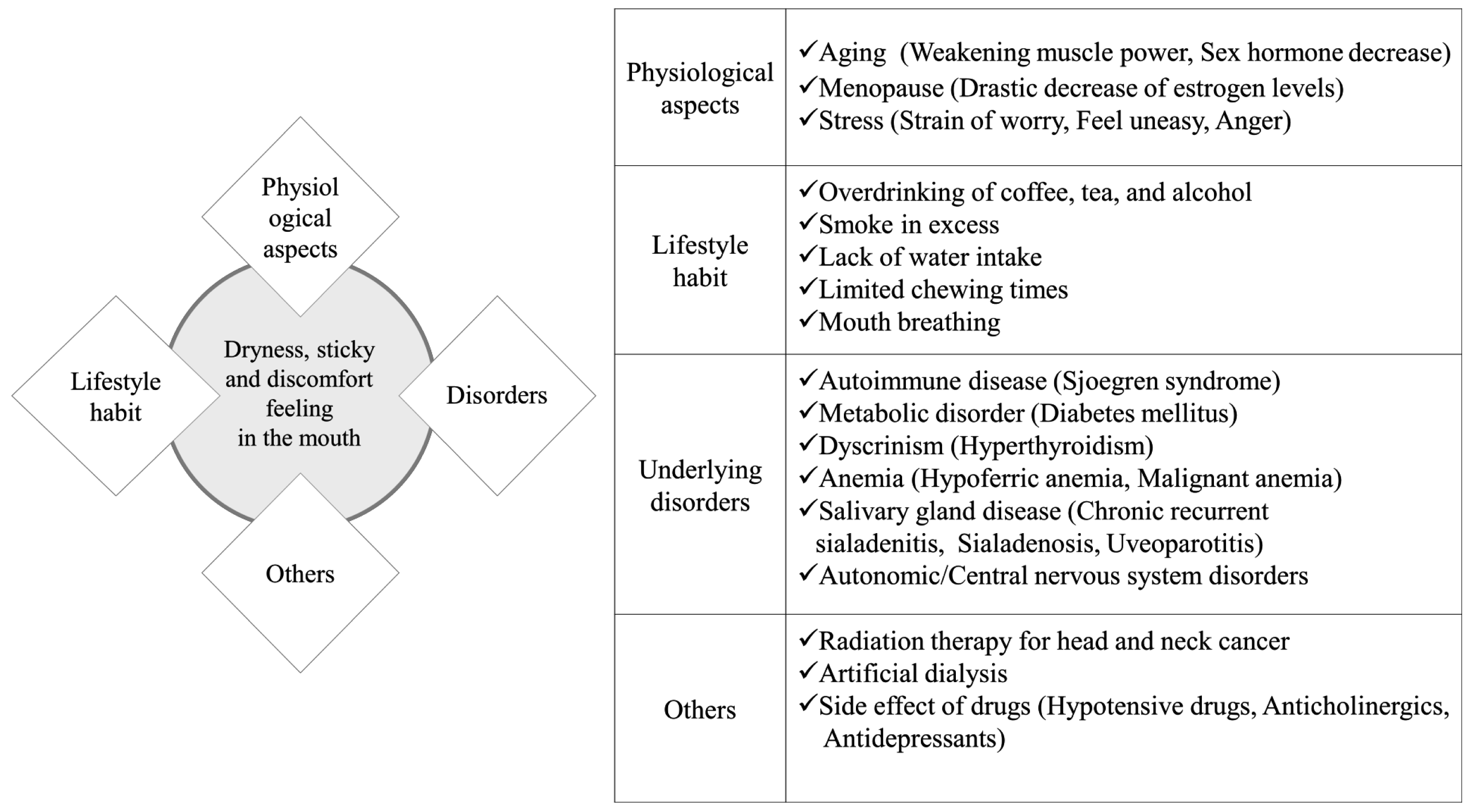

Supplement: Supplementary file 2 — Figure S1. Causes of mouth dryness. Mouth dryness is caused by various factors such as physiological, underlying disorders, and lifestyle habits. [file CRE2-5-7-s002.tif]

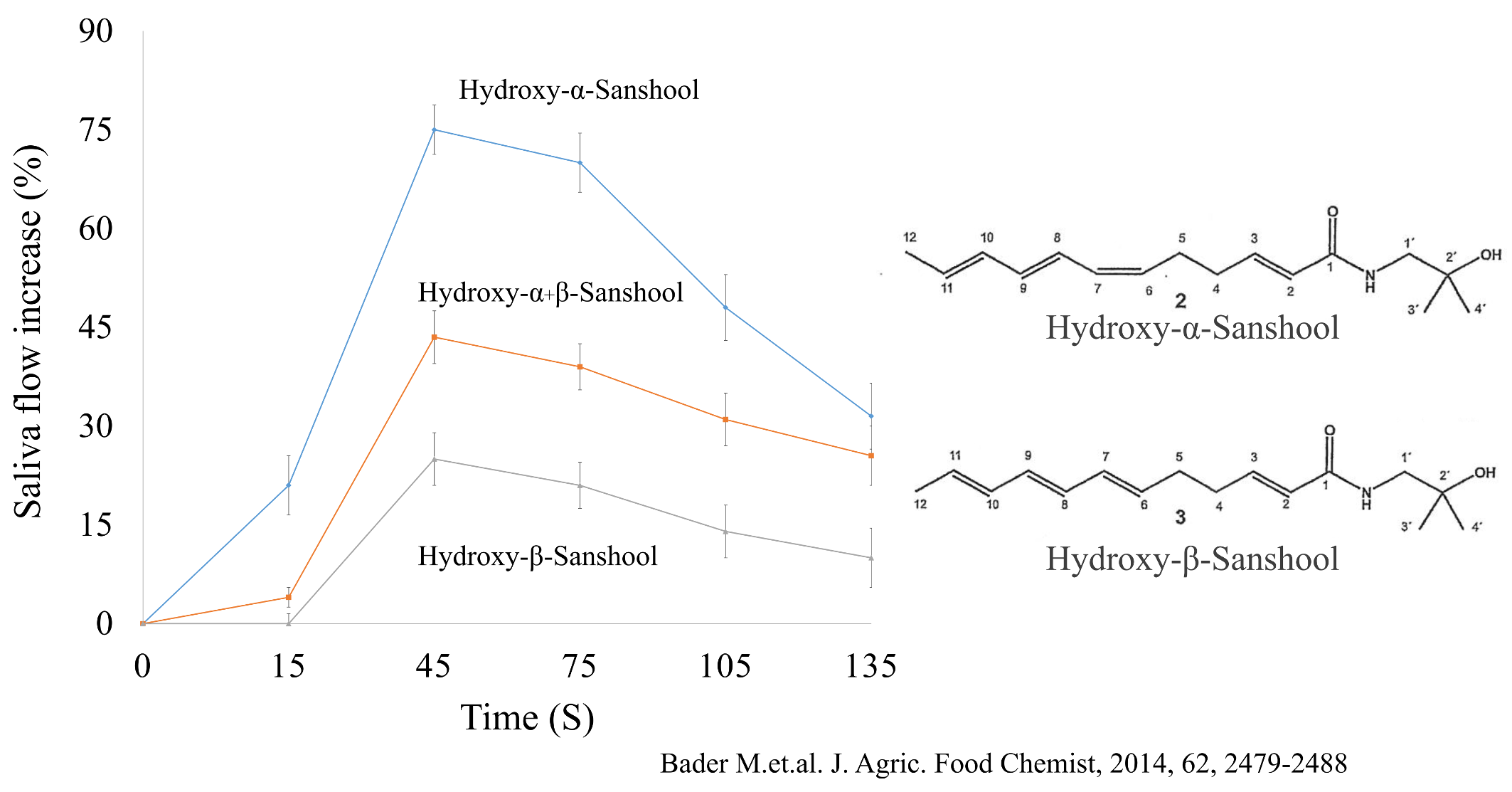

Supplement: Supplementary file 3 — Figure S2. The effect of hydroxy‐α‐sanshool on salivary secretion. The hydroxy‐α‐sanshool with at least one cis‐configured double bond induces a stronger salivation effect than the hydroxy‐β‐sanshool with all‐trans‐configured double bond (8 healthy volunteers). [file CRE2-5-7-s003.tif]

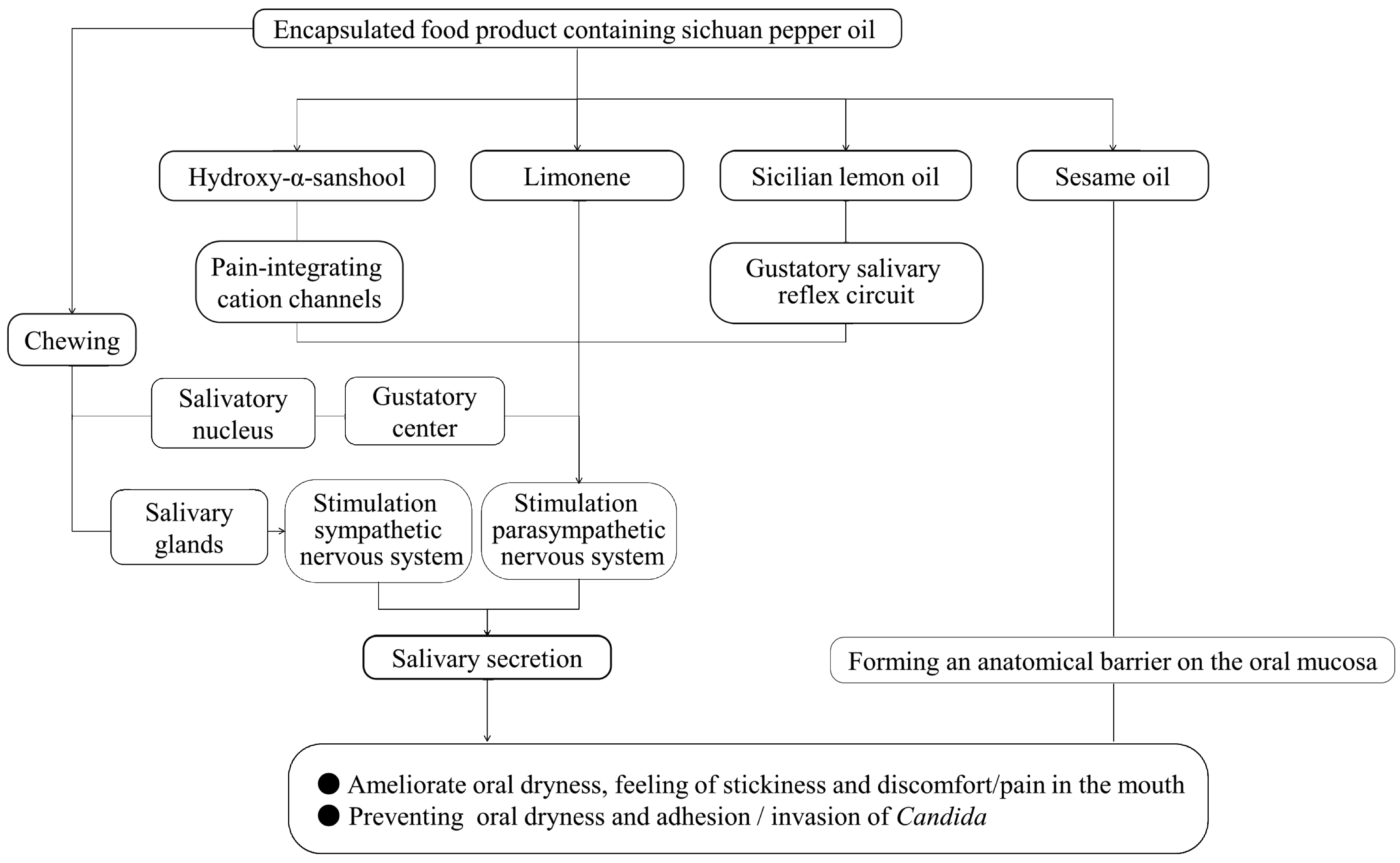

Supplement: Supplementary file 4 — Figure S3. Improvement in the dryness of the oral cavity (inference). When chewing the product, ingredients present in the product such as hydroxy‐α‐sanshool, limonene, and Sicilian lemon oil act comprehensively to promote salivary secretion. Chewing also leads to salivary secretion by stimulating the salivary glands. The sesame oil forms an anatomical barrier on the epithelium of the oral mucosa to prevent oral dryness and adhesion/invasion of Candida. Thus, the subjective symptoms, such as oral dryness, sticky sensation, and discomfort/pain in the mouth after ingestion are ameliorated quickly by the product. [file CRE2-5-7-s004.tif]
